# Clinical features and para-clinical findings of cryptococcal meningitis in the North of Iran during 2011-19

**DOI:** 10.18502/cmm.6.4.5330

**Published:** 2020-12

**Authors:** Farhang Babamahmoodi, Kobra Gerizade Firozjaii, Masomeh Bayani, Tahereh Shokohi, Jamshid Yazdani, Amir Mohammad Beyzaee, Fatemeh Ahangarkani

**Affiliations:** 1 Antimicrobial Resistance Research Center, Communicable Diseases Institute, Mazandaran University of Medical Sciences, Sari, Iran; 2 Infectious Diseases and Tropical Medicine Research Center, Health Research Institute, Babol University of Medical Sciences, Babol, Iran; 3 Invasive Fungi Research Center, Communicable Diseases Institute, Mazandaran University of Medical Sciences, Sari, Iran; 4 Department of Biostatistics, Faculty of Health, Mazandaran University of Medical Sciences, Sari, Iran

**Keywords:** Cryptococcal meningitis, Cryptococcus species, HIV, Iran

## Abstract

**Background and Purpose:**

Cryptococcal meningitis (CM) is a serious fungal infection that especially affects patients with human immunodeficiency virus (HIV). In this regard, the present retrospective study aimed to analyze the clinical and laboratory features and therapeutic outcomes of patients with CM admitted to two teaching referral centers in the north of Iran during 2011-19.

**Materials and Methods:**

This study was performed on all the hospitalized patients diagnosed with CM in two therapeutic centers of infectious diseases in the north of Iran. The required data, such as demographic characteristics and clinical and paraclinical features of patients, were extracted and entered in the information forms. Finally, the collected data were analyzed in SPSS software (version 16).

**Results:**

For the purpose of the study, records of 12 confirmed CM patients were evaluated in this research. Based on the results, 75% of the patients were male. Moreover, the average age of the subjects was 40.33± 8.93 years old and 66.6% of them (n=8) were HIV-positive. Other underlying diseases among HIV-positive patients included infection with hepatitis C virus (25%) and a history of tuberculosis (25%). In total, three HIV-negative patients suffered from Hodgkin lymphoma (25%), sarcoidosis (25%), and asthma (25%) and one patient (25%) had no underlying disease. Headache (75%), weakness, and fatigue (75%) were the most common symptoms among the participants. The cluster of differentiation 4 (CD4) count in all HIV-positive patients was less than 100 cells/μl. There was no significant difference between symptoms in HIV-positive and HIV-negative patients. Besides, no significant difference was observed between the groups of HIV-positive and HIV-negative patients regarding the period between the onset of symptoms and diagnosis of CM, the length of hospital stay, and the duration of antifungal medication consumption. In total, three patients (25%) expired, and six patients recovered. The CM recurred in two HIV-negative and one HIV-positive subjects; the two HIV-negative patients were treated, while the HIV-positive patient expired due to this recurrence.

**Conclusion:**

Clinical features and cerebrospinal fluid parameters were not different in HIV-positive and HIV-negative participants. Despite the fact that CM is not common in Iran, due to the increasing number of immunosuppressive patients, the differential diagnosis of CM should be considered for patients with signs and symptoms of infection in the central nervous system.

## Introduction

Cryptococcal meningitis (CM) is a serious fungal infection of the central nervous system that affects immunocompromised people, particul-arly patients with the human immunodeficiency virus (HIV). However, HIV-negative individuals, such as patients under immunosuppressive therapy and those with organic failure syndromes (e.g., post-organ transplant patients), diabetes mellitus, innate immune-logical disorders, common variable immunodeficiency syndrome, hematological disorders, liver cirrhosis, and alcoholism are at risk of this life-threatening infection [ 1 - 4 ]. Mortality rate of CM, even with antifungal therapy, is within the range of 30-50% which is considered high [ 5 ]. In addition to a high mortality rate, CM causes substantial morbidity [ 6 ].

Among numerous species of *Cryptococcus*, *C. neoformans* and *C. gattii* species complexes are the major causative agents of human cryptococcosis [ 7 ]. Measurement of cryptococcal antigen (Ag) titer and/or culture are the mainstay for a definite diagnosis. However, India ink staining of cerebrospinal fluid (CSF) is a sensitive test for the diagnosis of infection with *Cryptococcus* species. Accuracy of this method is reported to be more than 86% [ 2 ]. The clinical signs and symptoms of *Cryptococcus* species are indistinguishable from those of many other causes of meningitis; nevertheless, patients with CM have a slower onset of symptoms, compared to the patients infected with *Streptococcus pneumoniae* or *Neisseria meningitides*, since *Cryptococcus* progresses over days to a few weeks [ 8 ]. 

Presentation of CM can be acute or chronic with various symptoms, such as headache, fever, and nuchal rigidity suggesting meningeal irritation. As the clinical features are not specific, CM should always be considered in the differential diagnosis of chronic or sub-acute meningoencephalitis [ 8 ]. Patients typically experience fatigue, nausea, malaise, and impaired mental state for several weeks. The signs are frequently absent; however, they can include meningism, papilledema, cranial nerve palsies and other focal neurological defects, and abnormal level of consciousness [ 8 , 9 ]. Such complications, including increased intracranial pressure, are common and can cause profound visual or hearing loss in the absence of ventricular dilatation. However, patients suffering from obstructive hydrocephalus with ventricular dilation rarely experience cognitive impairment and gait ataxia [ 9 ]. 

In total, three classes of antifungal medications, namely the polyenes (amphotericin B), azoles (fluconazole and voriconazole), and a pyrimidine-derived drug (5-flucytosine) have been considered for optimal antifungal therapy of cryptococcosis [ 10 ]. It must be noted that 5-flucytosine is not available in some medical centers in Iran, while amphotericin B and fluconazole are routinely used for these patients. These agents may be used alone or in combination with other medications with varying degrees of effectiveness. 

There is a significant knowledge gap regarding the clinical epidemiological description of CM in Iran due to the lack of enough data from the surveillance epidemiological studies. Given the increasing number of immunocompromised patients in recent years and the increased risk of opportunistic fungal infections, such as CM, clinical epidemiology of this infection is critical for the healthcare system to optimize assessments in different areas, such as quality of care, patient safety, and health economics. The present retrospective study aimed to analyze the clinical and laboratory features and therapeutic outcomes of patients with CM admitted to two teaching hospitals in the north of Iran during 2011-19. 

## Materials and Methods

This cross-sectional retrospective study was performed in two tertiary therapeutic centers of infectious diseases in the north of Iran, namely Razi and Rohani hospitals. This study was approved by the Ethics Committee of Mazandaran University of Medical Sciences (code: IR.MAZUMS. REC.1397.3216). It must be mentioned that the samples were selected using the census sampling method. The study population included all patients with CM whose initial identification of *Cryptococcus* species in CSF had been confirmed by direct microscopic examination via India ink and positive culture. 

The required data, including demographic characteristics, clinical and paraclinical features, risk factors, and medical history of the patients were collected and entered in the data forms. Afterward, the collected data were analyzed in SPSS software (version 16). Furthermore, the descriptive data are presented as numbers and percentages. The differences between the groups were determined using the chi-square test or Fisher's exact test. It must be noted that a *P-value* of less than 0.05 was considered statistically significant. 

## Results

**Study population, demographic characteristics, risk factors, and clinical features**

In this study, records of 12 confirmed CM patients were evaluated. In total, 75% (n=9) of the participants were male and the rest were female. Moreover, the subjects were within the age range of 26-55 years old and their mean age was 40.33± 8.93 years old. The demographic characteristics, clinical features, and risk factors of patients based on their HIV positive or negative status are summarized in Table 1. Based on the findings, 66.6 % (n=8) of the patients were HIV-positive. The underlying diseases among HIV-positive patients were hepatitis C virus infection (n=2, 25%) and a history of tuberculosis (n=2, 25%). 

**Table 1:** Demographic characteristics and clinical features of patients

Demographic and Clinical Characteristics	Variables	HIV Positive	HIV Negative	P
		n=8	n=4	
Demographic characteristics	Age (Mean± SD) years	38±5.782	45±13.089	0.57
	Male	6 (75%)	3 (75%)	0.67
	Female	2 (25%)	1 (25%)	0.60
Underlying Status	Hepatitis C virus	2 (25%)	0	2
	Hodgkin lymphoma	0	1 (25%)	1
	Sarcoidosis	0	1 (25%)	1
	History of Tuberculosis	2 (25%)	0	1
	Asthma	0	1 (25%)	0.25
	Brain Shunt Implantation	0	1 (25%)	0.25
	History of cryptococcal meningitis	1 (12.5%)	0	01
Sign and Symptoms	Emesis	4 (50%)	1(25%)	0.576
	Photophobia	1 (12.5%)	1(25%)	1
	Seizure	5 (62.5%)	0	0.081
	Ataxia	2 (25%)	1 (25%)	1
	Constipation	1 (12.5%)	1(25%)	1
	Muscle spasm	1 (12.5%)	0	1
	Fever	4 (50%)	2 (50%)	1
	Chill	3 (37.5%)	2 (50%)	1
	Headache	6 (75%)	3 (75%)	1
	Urine incontinency	2 (25%)	2 (50%)	0.55
	Painful eye ‎movement	1 (12.5%)	1 (25%)	1
	Drowsiness	2 (25%)	2 (50%)	0.55
	Behavioral changes	3 (37.5%)	0	0.49
	Vertigo	1 (12.5%)	2 (50%)	0.49
	Blindness	2 (25%)	0	0.51
	Blurred vision	2 (25%)	2 (50%)	0.55
	Dyspnea	1 (12.5%)	0	1
	Weakness and fatigue	7 (87.5%)	2 (50%)	0.49
	Cough	5 (62.5%)	1 (25%)	0.55
	Reduce muscle force	1 (12.5%)	1 (25%)	1
	Oculomotor nerve palsy	1 (12.5%)	0	1
	Abducens nerve palsy	1 (12.5%)	0	1
Other fungal infections	Oral candidiasis	4 (50%)	0	0.2
Period between the onset of symptoms and diagnosis (Mean± SD) days		16±22.716	18.25±11.73	0.495
Duration of hospitalization (Mean± SD) days		29.13±7.338	22.75±13.745	0.592
Duration of antifungal treatment (Mean± SD) days		24.38±7.4	16.5±15.94	0.549

In total, three HIV-negative patients suffered from underlying diseases, such as Hodgkin lymphoma (n=1, 25%), sarcoidosis (n=1, 25%), and asthma (n=1, 25%), while one patient had no underlying disease. Headache (75%), weakness and fatigue (75%), cough and sputum (50%), fever (50%), chills (41.7%), emesis (41.7%), and seizures (41.7%) were the most common symptoms among patients. In addition, the most frequent symptoms in HIV-positive patients included weakness, headache, cough, and seizures, while the most common symptom in HIV-negative patients was a headache. 

There was no significant difference between symptoms in HIV-positive and HIV-negative patients. Moreover, there were no significant differences between the groups of HIV-positive and HIV-negative patients in terms of the period between the onset of symptoms and diagnosis of CM (*P*=0.495), the length of hospital stay (*P*=0.552), and the duration of antifungal medication consumption (*P*=0.549). 

**Para-Clinical characteristics**

The computed tomography scan was performed for six patients. Hydrocephalus and parenchymal lesion were seen in one patient, while the results of the computed tomography of other patients were normal. In addition, magnetic resonance imaging (MRI) was performed for eight patients. Based on the MRI results, two (25%) and three (37.5%) patients had hydrocephalus and parenchymal lesions, respectively, while three patients (37.5%) were normal. 

Mean value of the cluster of differentiation 4 (CD4) count in HIV-positive patients was 48.667±23.76/µ. Table 2 tabulates the laboratory findings of CM patients based on their HIV positive or negative status. There were no significant differences between HIV-positive and HIV-negative patients regarding the results of their CSF examination. Direct examination of CSF of all patients with India ink revealed numerous encapsulated yeast cells which are suggestive of the presence of *Cryptococcus* species. The HIV-positive patients had a lower WBC count in serum compared to HIV-negative patients, which was statistically significant (*P*=0.048). 

**Table 2:** Laboratory results of cryptococcal meningitis patients

	Variables	HIV Positive (n=8)	HIV Negative (n=4)	*P*
**CSF Examination**	WBC cells/mm3 (median± IQR)	207.75±421.006	302.50±364.452	0.214
	RBC cells/mm3 (median± IQR)	912.50±2459.871	15±23.805	0.073
	PMN	35.71±33.094	41.25±37.722	0.788
	lymphocytes	64.29±33.094	58.75±37.722	0.788
	LDH	65.29±24.479	65.50±37.477	0.889
	Protein	63.13±24.439	116.75±70.934	0.283
	Glucose	35.62±15.14	31.75±22.277	0.570
	Positive Indian ink	100%	100%	
	Positive culture	37.5%	50%	
Serum Examination	WBC cells/mm3 (median±IQR)	4750±1764.734	7000±1363.818	0.048
	RBC cells/mm3 (median±IQR)	3746250±948456.453	4417500±176517.232	0.570
	Platelet cells/mm3 (median±IQR)	170500±91832.145	211750±81147.499	0.461
	HB	11.413±2.1768	11.575±2.5513	0.933
	MCV	87.25±8.681	76±10.893	0.154
	MCH	28.5±3.625	25.75±4.992	0.461
	Sodium	135.63±7.482	133.5±3.416	0.933
	Potassium	3.787±0.8887	3.45±0.5745	0.570
	ESR	69±31.505	53.5±22.67	0.461
**Specific Examinations for HIV positive patients**	HIV viral load (n/N) IU/ml Mean± SD (minimum-maximum)	4/8 888540±1204921 (32520-2671080 )	NA
	CD4 count/microliter	48.667±23.76	NA

CSF: cerebrospinal fluid, IQR: interquartile range, WBC: White blood cells, RBC: Red blood cell, PMN: Polymorphonuclear, LDH: Lactate dehydrogenase, HB: Hemoglobin, MCV: Mean corpuscular volume, ESR: Erythrocyte sedimentation rate, NA: not applicable

**Antifungal treatment and outcome**

Management of CM was divided into three phases: induction, consolidation, and maintenance therapy. Induction therapy was given to the patients with the aim of rapid sterilization of cerebrospinal fluid. The induction regimen included amphotericin B deoxycholate (0.5-1.0 mg/kg per day) and flucytosine (100 mg/kg per day) based on the availability of the medications. A lumbar puncture and culture should be performed two weeks after the treatment to demonstrate CSF sterility. 

Consolidation regimen consisted of fluconazole for both HIV-negative (400 mg daily) and HIV-positive (400-800 mg daily) patients for 8-10 weeks following the induction phase. After successful induction and consolidation therapy, culture-negative HIV-positive patients received fluconazole (200 mg/day) for maintenance therapy. The antifungal treatment regimen for HIV-positive and HIV-negative groups was based on the availability of standard medication choices. 

Briefly, eight patients received the mean dosage of 59.4 (50-75) mg amphotericin B for the mean duration of 14.75 days (2-34 days). Four of these patients received liposomal amphotericin B from the beginning and three patients who were initially on amphotericin B deoxycholate treatment were switched to liposomal amphotericin B due to the side effects, such as acute kidney injury, anemia, and hypokalemia. The treatment side effects were not significantly different (*P*=0.97) between HIV-positive and HIV-negative patients. Mean duration of using liposomal amphotericin was 15.7 days (3-30 days) with a mean dosage of 200 (50-400) mg. 

In total, three patients received flucytosine from the beginning of treatment while for one patient flucytosine was added to the treatment regimen during treatment. Mean duration of using flucytosine was 18 days (8-23 days) with the mean dosage of 4500 (6000-2000) mg. In addition, one patient received voriconazole during treatment for the mean duration of nine days, and the mean dosage of 800 mg. All patients received fluconazole for the mean duration of 20.8 days (2-37 days) and the mean dosage 629.167 (1000-200) mg in their treatment regimen. 

In total, three patients (25%) expired, two of whom (66%) were HIV-positive and the other one had an underlying risk factor, i.e., brain shunt implantation The mean period between hospitalization and anti-fungal prescription of deceased and survived patients were 8.6±10 and 3.2±3.3 days, respectively (*P*=0.373). The survived patients were followed up for three months after discharge from the hospital and it was found out that six of them recovered. Moreover, the recurrence of CM was observed in three patients (two HIV-negative and one HIV-positive); two of them were treated and one of them (HIV-positive) expired due to this recurrence. 

**Comparisons of laboratory results of deceased and survived patients**

Among serum and CSF factors, Polymorphonuclear (PMN) and lymphocytes count in CSF had significant associations with mortality (*P*=0.048). Table 3 summarizes the comparison of laboratory results of deceased and survived patients. 

**Table 3:** Laboratory findings of deceased and survived patients

	Variables	Deceased (n=3)	Survivor (n=9)	*P*
CSF Examination	WBC	276±453	226±394	1
	RBC	26±25	808±2321	0.6
	PMN	71±12	25±29	0.048
	lymphocytes	28±12	75±29	0.048
	LDH	67±7.07	64.86±28.70	0.667
	Protein	60.6±28.3	87.78±54.41	0.373
	Glucose	42±24.3	31.78±14.64	0.727
Serum Examination	WBC	6633±1871	5122±1895	0.282
	RBC	4696666±275378	3727777±816100	0.064
	Platelet	189666±97161	182444±89670	1
	HB	13.4±1.8	10.8±1.9	0.1
	MCV	83±7.5	83.5±11.7	1
	MCH	28±5	27.3±4	0.727

WBC: White blood cells, RBC: Red blood cell, PMN: Polymorphonuclear, LDH: Lactate dehydrogenase, HB: Hemoglobin, MCV: Mean corpuscular volume, MCH: Mean corpuscular hemoglobin

## Discussion

Studies conducted on CM in Iran are limited to case reports [ 1 , 11 ]. Regarding the lack of enough data in this regard, this retrospective study aimed to evaluate the clinical epidemiology of patients with CM during eight years in the north of Iran. The CM is among the most serious infections and is particularly observed in HIV-positive patients. In the present study, 91.6% of patients suffered from the predisposed condition for CM as 66.6 % of patients were HIV-positive. Furthermore, the HIV-negative patients were predisposed to risk factors, such as malignancy, sarcoidosis, and brain shunt implantation. However, one patient did not have a history of underlying diseases. 

This finding is consistent with those of a study conducted by Lee et al. who found that two HIV-negative patients had no risk factors. Besides, this finding is in line with that of the case report performed by Ghasemian et al. about an immunocompromised patient with CM. Therefore, in patients with symptoms of meningitis, CM should be included in the list of differential diagnoses, regardless of whether or not they have relevant underlying diseases or risk factors [ 12 , 13 ]. In HIV-positive patients, CM is associated with profound immunosuppression, usually occurring at CD4 counts less than 100 cells/μl [ 13 ]. In accordance with the established facts, in the present study, the CD4 count in all HIV-positive patients was less than 100 cells/μl. 

Findings of this research demonstrated some differences between HIV-positive and HIV-negative patients. However, in the present study, there was no significant difference between these two groups regarding the period between the onset of symptoms and hospitalization. In this study, it was found that this period in HIV-positive patients was shorter, in comparison to the HIV-negative patients which is consistent with the results of the study performed by Liao et al. [ 14 ]. 

Moreover, in the present study, the most common symptoms included headache, fever, weakness, lethargy, cough, and sputum. This was partially in line with the results of a research conducted by Zhang et al. [ 15 ] who reported headache, fever, and emesis as the most common symptoms. Besides, this is consistent with the findings of the study carried out by Lee et al. who mentioned headache, fever, and altered state of consciousness as the most common symptoms [ 12 ]. The slight differences between the findings of the present and the above-mentioned studies can be due to the heterogeneity of this study with those two in terms of the number of HIV patients. 

In the present study, the level of the leukocyte in the CSF of HIV-positive patients was lower than that of the HIV-negative individuals which were consistent with the findings of the studies carried out by Lee et al. and Liao et al. [ 12 , 14 ]. Nevertheless, in the present study, these differences were not statically significant which may be due to its small statistical population. In this study, HIV-negative subjects had higher WBC levels of serum, compared to HIV-positive patients (*P*=0.048). 

Similarly, the results of other studies indicated that the serum leukocyte levels were significantly lower in HIV-positive patients, compared to HIV-negative subjects [ 12 , 16 ]. However, this finding needs to be confirmed by further studies performed on larger statistical populations. In this study, there were no significant differences between HIV-positive and HIV-negative subjects regarding most of the laboratory findings as the small sample size might have led to the underestimation of some of the significant factors. 

In the present study, the mean duration of antifungal therapy of HIV-positive patients was longer, compared to that of the HIV-negative subjects. This can be due to the immunodeficiency status of HIV-positive patients. Generally, amphotericin-B-based therapy is the first choice for the treatment of CM [ 10 , 17 ]. In this study, Amphotericin B (Deoxycholate or Liposomal form) and fluconazole were the medication of choice for all the patients while flucytosine and voriconazole were added to the treatment of four and one patients, respectively. 

It must be noted that the higher proportion of predisposing factors and severe side-effects may cause clinicians to choose or change the antifungal regimen. Mortality rate of CM in HIV-positive patients is still high, even in developed countries [ 9 ]. In this study, 25% of patients expired, 66.66% of whom were HIV-positive. Bandalizadeh et al. in their study reported the mortality rate of CM to be 64%. The higher mortality rate in the study of Bandalizadeh et al. may be due to the more severe immunodeficiency condition of patients and also the lack of access to the use of flucytosine in the first-line treatment in combination with amphotericin B [ 7 ]. 

In this study, the rate of mortality had a significant relationship with the high percentage of PMN and low level of lymphocytes in CSF. However, the glucose level of CFS was not associated with the increase in the mortality rate. On the other hand, Zhang et al. reported decreased glucose concentration of CSF as an effective factor on the increase of mortality [ 15 ]. Generally, it was not possible to compare the findings of this research with those of other studies due to the small population of the present study. 

There are also some limitations in this research due to its retrospective design. Accordingly, the required data were collected from patient medical records and laboratory data; however, some records were misclassified or some data were missing. Other drawbacks of our study were its small sample size, lack of species identification, and lack of the required data to compare HIV-positive and HIV-negative patients regarding the cryptococcal antigen titer. 

## Conclusion

In conclusion, the clinical features and CSF parameters of HIV-positive and HIV-negative participants were not different. Despite the fact that CM is not common in Iran, the increasing number of immunosuppressive patients requires the differential diagnosis of CM for patients presenting with signs and symptoms of infection in CNS. 

## Authors’ contribution

In this research, F. B, K. GF, M. B, T. S, J. Y, AM. B and F. A designed the study, acquiesced, analyzed, and interoperated the data. All the authors participated in the preparation of the draft of the manuscript and approved its final version.

## Financial disclosure

None Declared.
